# Bicarbonate-Based Solution for the Management of Established Acute Kidney Injury: A Pilot Open-Label Observation Study

**DOI:** 10.7759/cureus.42127

**Published:** 2023-07-19

**Authors:** Rolando Claure-Del Granado, Vania C Prudencio-Ribera, Vineet Gupta, Jason Yang, Kianoush Kashani, Rakesh Malhotra

**Affiliations:** 1 Division of Nephrology, Department of Medicine, Hospital Obrero No. 2 – Caja Nacional de Salud (CNS) Universidad Mayor de San Simon, Cochabamba, BOL; 2 Pulmonology, University Hospital of Toledo, Toledo, ESP; 3 Medicine/Hospital Medicine, University of California San Diego School of Medicine, San Diego, USA; 4 Medicine, University of California San Diego School of Medicine, San Diego, USA; 5 Pulmonary and Critical Care Medicine, Mayo Clinic, Rochester, USA; 6 Nephrology and Hypertension, Mayo Clinic, Rochester, USA; 7 Division of Nephrology and Hypertension, Department of Medicine, University of California San Diego School of Medicine, San Diego, USA

**Keywords:** acidosis, intravenous fluids, chloride-rich, normal saline, bicarbonate-free solutions, bicarbonate-based solutions, acute kidney injury

## Abstract

Background

Administration of intravenous (IV) solutions constitutes a key component of acute kidney injury (AKI) management. However, the optimal IV fluid solution in the setting of AKI remains uncertain. In this study, we assessed whether the use of bicarbonate-containing solution in patients with established AKI is associated with early renal recovery as compared to bicarbonate-free solutions.

Methods

We performed an open-label observational pilot study in 59 patients with established AKI. IV fluid solutions that were used include bicarbonate-based solution with low chloride content (80 mEq/L of 8% sodium bicarbonate in a solution that contains 77 mEq/L of sodium, 77 mEq/L of chloride and 25 g/L of glucose) or solutions without bicarbonate with high chloride content (0.9% normal saline, 0.45% half-saline, normal ringer, or 4% succinylated gelatine). We evaluated the association of IV fluids type with renal recovery.

Results

The median age of study participants was 66 years (inter-quartile range (IQR) 37-85), and 59% (n=35) were men. The prevalence of diabetes and chronic kidney disease (CKD) stages 1-3 were 34% (n=20) and 39% (n=23), respectively. Patients who received bicarbonate-based IV solutions had a greater reduction of serum creatinine (sCr) per day (delta sCr) as compared with patients who received bicarbonate-free solutions (-0.29±0.47 vs. 0.07±0.42; p=0.007). The renal recovery was faster in patients who received bicarbonate-based solutions as compared to the bicarbonate-free group (days from peak sCr to baseline sCr: 5.6±2.1 vs. 7.6±2.8; p < 0.001, respectively).

Conclusions

We observed faster renal recovery in patients with established AKI who received the bicarbonate-based solution with low chloride content. Our study findings require confirmation in larger cohorts.

## Introduction

Acute kidney injury (AKI) is a frequent, life-threatening disease that occurs as a consequence of a broad range of clinical conditions. AKI is independently associated with increased morbidity, mortality, and length of hospital stay [1,2]. AKI management relies on several key strategies, including the identification of patients who are susceptible to developing AKI, such as those experiencing volume depletion. It is crucial to reduce exposure to factors that could potentially cause AKI, such as nephrotoxic drugs or radiocontrast agents. Additionally, providing supportive care is essential, while also addressing acute complications such as hyperkalemia, metabolic acidosis, and fluid overload. Volume depletion is a known risk factor for AKI, and fluid resuscitation might help to halt the progression of AKI and could facilitate the recovery of kidney function. However, the type and amount of intravenous (IV) fluid that should be used in AKI patients is still a matter of debate.

Most of the discussion about the optimal fluid strategy to be used in patients with AKI has focused on crystalloids versus colloids. Current guidelines advocate the use of isotonic crystalloids rather than colloids as the initial fluid resuscitation therapy in patients at risk of AKI or with established AKI and in patients with sepsis [3]. However, recent studies have shown that crystalloid solutions with high chloride content are linked with an increased risk of occurrence of AKI [4,5]. Possible mechanisms of chloride-associated kidney outcomes are due to the activation of the tubuloglomerular feedback mechanism, which results in increased afferent arteriolar vasoconstriction, mesangial contraction, and associated reduction in glomerular filtration rate (GFR) [6]. In addition, hyperchloremia may induce thromboxane release with coupled vasoconstriction, as well as enhanced responsiveness to vasoconstrictor agents as shown in an animal model [7]. Chloride-rich crystalloid solutions can also lead to metabolic acidosis that is related to interleukin production (Interleukin 6, interleukin 10, and tumor necrosis factor) and insulin resistance that can cause harmful effects on kidney function [8]. Furthermore, patients with AKI often develop metabolic acidosis, which is associated with a number of adverse effects that are harmful to kidney function. Despite the presence of metabolic acidosis, sodium bicarbonate administration to prevent AKI or to improve renal function has not been fully studied. Moreover, the benefits of alkalinization of fluids in established AKI have been less explored. In this study, we aim to evaluate if the use of a bicarbonate-based solution with low chloride content versus the solutions with high chloride content and without bicarbonate could result in early renal recovery in patients with established AKI.

## Materials and methods

We performed a prospective, open-label observational pilot study at the Hospital Obrero No 2 - Caja Nacional de Salud (C.N.S.), a tertiary care hospital affiliated with the Universidad Mayor de San Simon, School of Medicine (Cochabamba, Bolivia). No informed consent was required from patients since different solutions used are part of a protocol-based solution management at the Medicine Department of our Institution. The human research ethics committee of the Hospital Obrero No 2 - C.N.S., approved the study with a waiver for informed consent.

Subjects

Over the period of six months, 59 consecutive hospitalized patients who developed AKI were enrolled in the study. AKI was defined as a rise in serum creatinine (sCr) by equal or higher to 0.3 mg/dl within 48 hours, or an increase of >1.5 times the reference sCr as per the Kidney Disease Improvement Global Outcomes (KDIGO) criteria. Exclusion criteria include patients with chronic kidney disease (CKD) stages 4 and 5 defined by the CKD-Epidemiology Collaboration (CKD-EPI) equation, presence of kidney allograft, and those who received any type of renal replacement therapy (RRT). We further excluded patients who developed contrast-associated AKI (CA-AKI). The reason for exclusion was that our institution has an IV fluid protocol for the prevention of CA-AKI, which include IV hydration with 850 ml of 5% dextrose + 3 ampules of 50 mmol sodium bicarbonate before and after the study.

A reference creatinine was determined to assess the diagnosis of AKI at enrollment in the descending order of preference: (1) the most recent pre-enrollment sCr value between three months and 365 days; and (2) pre-hospital sCr value between admission and three months.

CKD was defined using KDIGO recommendations as either an evidence of kidney damage >3 months (urine albumin to creatinine ratio equal or higher than 30 mg/g, proteinuria >150 mg in 24 hours or abnormalities on ultrasound) or the presence of estimated glomerular filtration rate (eGFR) <60 mL/min/1.73 m^2^ for >3 months, with or without other signs of kidney damage [9]. CKD was staged based on the level of calculated EPI-CKD eGFR. Baseline sCr to determine CKD status was the lowest measured or historical value within >3 months of hospital admission. Kidney recovery was adjudicated when sCr returned to its baseline value or if the sCr levels were higher than baseline value but within equal or lower 0.3 mg/dL times baseline levels at day 7 after AKI diagnosis was made [10].

IV fluids intervention

All patients included in the study required IV fluids therapy and were either in one of the phases of the conceptual model of fluid resuscitation (Rescue, Optimization, Stabilization, or De-escalation (ROSD)) [11]. Briefly, the rescue phase included rapid boluses (infusion of IV fluid of at least 500 ml over 15 minutes). In the optimization phase, the fluid infusion we used included 100-200 ml bolus of IV fluids over 5-10 min with the re-evaluation of tissue perfusion. During the stabilization phase, minimal maintenance infusion was used with no more than 1-2 ml/kg/h of IV fluids in case of inadequate oral fluid intake. Depending on clinical signs and response, the treating physician selected the initial and subsequent type of IV solutions (either the bicarbonate-based solution with low chloride content or the various solutions with high chloride content and without bicarbonate), as well as volume and the rate of administration. The IV fluid solutions of choice were 1) 0.9% normal saline, 0.45% half saline, normal ringer, and Gelfusine (Table 1 shows the composition of the different types of solutions that were employed) for bicarbonate-free, high-chloride solutions, and 2) pharmacist-prepared, bicarbonate-based solution with low chloride content by mixing 80 mEq/L of 8% sodium bicarbonate in a solution that contains 77 mEq/L of sodium, 77 mEq/L of chloride and 25 g/L of glucose (“Solucion Glucosalina”; Drogueria INTI S.A. La Paz, Bolivia). The latter solution is used as an alternative to Plasma-Lyte 148, which is a balanced solution with low chloride content (98 mmol/L of chloride, Baxter Healthcare, Deerfield, IL, USA).

**Table 1:** Composition of bicarbonate-free solutions used in the study Na^+^, sodium; K^+,^ potassium; Cl^-^, chloride.

	Na+ (mEq/L)	K^+^ (mEq/L)	Cl^-^ (mEq/L)	Ca^++ ^(mEq/L)	Glucose (g/L)
0.9% Normal Saline	153.85	0	153.85	0	0
0.45% Half Saline + Glucose	77.0	0	77.0	0	25.0
Normal Lactate	147.0	4.0	155.5	4.5	0
Gelfusine	154.0	0	120.0	0	0

Outcomes

During the hospital stay, sCr was monitored at least every 24 hours, and urine output was monitored every hour, with other venous blood chemistry and electrolytes measurements requested at the discretion of the treating clinician. We assessed the effect of IV fluid type in AKI recovery by calculating changes in serum creatinine (delta sCr), urine output, the number of days it took for the sCr value to return to its baseline, and finally, the amount of solutions that was administered in each group of patients.

Statistical analysis

Continuous variables were expressed as the mean ± standard deviation (SD) or median and inter-quartile range (IQR). Between-group comparisons of continuous variables were performed using the independent t-test or Mann-Whitney U test, after testing for normality using the Kolmogorov-Smirnov test. Dichotomous variables were compared using the chi-square test or Fisher’s exact test. All statistical analyses were two-tailed and performed using SPSS 20 software (IBM Corp., Armonk, NY, USA). The two-sided P <0.05 was considered statistically significant.

## Results

The baseline characteristics of the study population are shown in Table 2. No differences were found in the baseline eGFR, urine output, and AKI etiology between the two study groups (Table 2). CKD was present in 13 (44.8%) of the 29 patients who received bicarbonate-based solution with 30.8% of patients having CKD at stage 2 and 69.2% having stage 3 CKD. Whereas 10 (33.2%) of patients who received IV fluid solutions without bicarbonate had CKD, with 30% of them having stage 2 CKD and 70% having stage 3 CKD. No adverse events were reported in either of the two groups.

**Table 2:** Baseline characteristics of the study cohort IQR, interquartile range; AKI, acute kidney injury; CKD, chronic kidney disease; CVD, cardiovascular disease; COPD, chronic pulmonary obstructive disease; CRS, cardiorenal syndrome; HRS, hepatorenal syndrome; eGFR, estimated glomerular filtration rate; MDRD, modification of diet in renal disease; CKD-EPI, chronic kidney disease epidemiological collaboration.

Characteristics	Bicarbonate-Based Solution Group (N = 29)	Bicarbonate-Free Solution Group (N = 30)
Age, years median (IQR)	64 (37-85)	67 (37-97)
Gender, male, n (%)	21 (72)	14 (47)
Physiological variables		
Mean arterial pressure, mmHg	83	84
Heart rate, beats/min	77	76
Respiratory rate/min	20	19
Urine output, ml/hr	66	58
Comorbidities, n (%)		
Diabetes	9 (31)	11 (37)
Hypertension	8 (28)	9 (30)
Cardiac failure	9 (31)	9 (30)
Liver disease	2 (7)	2 (7)
CKD stages 1-3	13 (45)	10 (33)
Obesity	2 (7)	2 (7)
Obstructive uropathy	1 (3)	2 (7)
Comorbid burden		
0	9 (31)	6 (20)
1	6 (21)	8 (27)
2	6 (21)	9 (30)
≥3	8 (28)	7 (23)
AKI Etiology, n (%)		
Pre-renal	6 (21)	5 (17)
Obstructive nephropathy	1 (3)	1 (3)
Sepsis	16 (55)	16 (53)
CRS	3 (10)	3 (10)
HRS	1 (3)	0 (0)
Rhabdomyolysis	1 (3)	1 (3)
Multifactorial*	1 (3)	4 (13)
eGFR - ml/min per 1.73 m^2^		
MDRD	27	33
CKD-EPI	26	31

Baseline sCr at admission was higher in patients who received the bicarbonate and low chloride content (77 mEq/L) solution when compared with patients who received bicarbonate-free solutions and high chloride content (120-155 mEq/L) (median (IQR) 1.12 mg/dL (IQR 0.9-1.3) vs. 1.08 mg/dL (0.9-1.23); p < 0.001) (Table 3). In the bicarbonate-based solution group, seven (24.1%) patients developed stage 1 AKI; 14 (48.3%) patients developed stage 2 AKI; and eight (27.6%) patients developed stage 3 AKI. In the bicarbonate-free solution group, 14 (46.7%) patients developed stage 1 AKI; 14 (46.6%) patients developed stage 2 AKI; and two (6.7%) patients developed stage 3 AKI. Peak sCr was higher in the group of patients who received the bicarbonate-based solution (mean±SD, 3.09±1.33 mg/dL vs. 1.96±0.38 mg/dL; p < 0.001) (Table 3). None of the patients required kidney replacement therapy.

**Table 3:** Renal recovery and length of hospital stay between bicarbonate-based and bicarbonate-free solutions IQR, interquartile range; SD, standard deviation; sCr, serum creatinine

	Bicarbonate-Based Solution (n=29)	Crystalloid Solutions (n=30)	P value
Median (IQR) baseline sCr (mg/dL), before AKI	1.12 (0.9-1.3)	1.08 (0.9-1.23)	< 0.001
Mean (±SD) peak AKI sCr (mg/dL)	3.09 ± 1.33	1.96 ± 0.38	< 0.001
Mean ± SD D sCr (mg/dL)	- 0.29 ± 0.47	- 0.07 ± 0.42	0.007
Median (IQR) urine output (mL)	1592 (1409-1905)	1647 (1296-2192)	0.29
Median (IQR) of volume of solutions used (mL/per day)	1000 (500-2000)	1000 (1000-2000)	0.90
Mean±SD of days for sCr to return to baseline value	5.6 ± 2.1	7.6 ± 2.8	< 0.001
Median (IQR) of duration of hospital stay (days)	7.65 (4-12)	9.4 (4-14)	< 0.001
In-hospital mortality (%)	1 (3.4%)	4 (13.3%)	< 0.001

**Figure 1:**
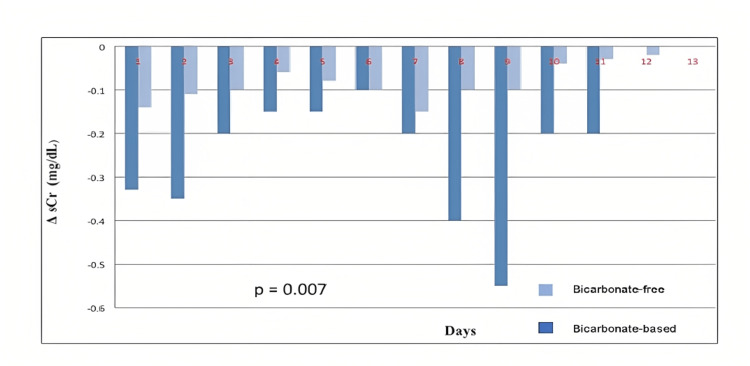
Delta serum creatinine (sCr) by day in each of the study group Delta serum creatinine (sCr) by day in each of the study group; dark blue box represents patients that received bicarbonate-based solutions, and light blue box represents patients that received bicarbonate-free solutions.

The reduction of sCr per day (delta sCr) was greater in patients who received the bicarbonate-based solution with the low chloride content as compared with patients who received bicarbonate-free solutions (mean±SD, -0.29±0.47 vs. 0.07±0.42; p = 0.007, respectively) (Figure 1).

**Figure 2:**
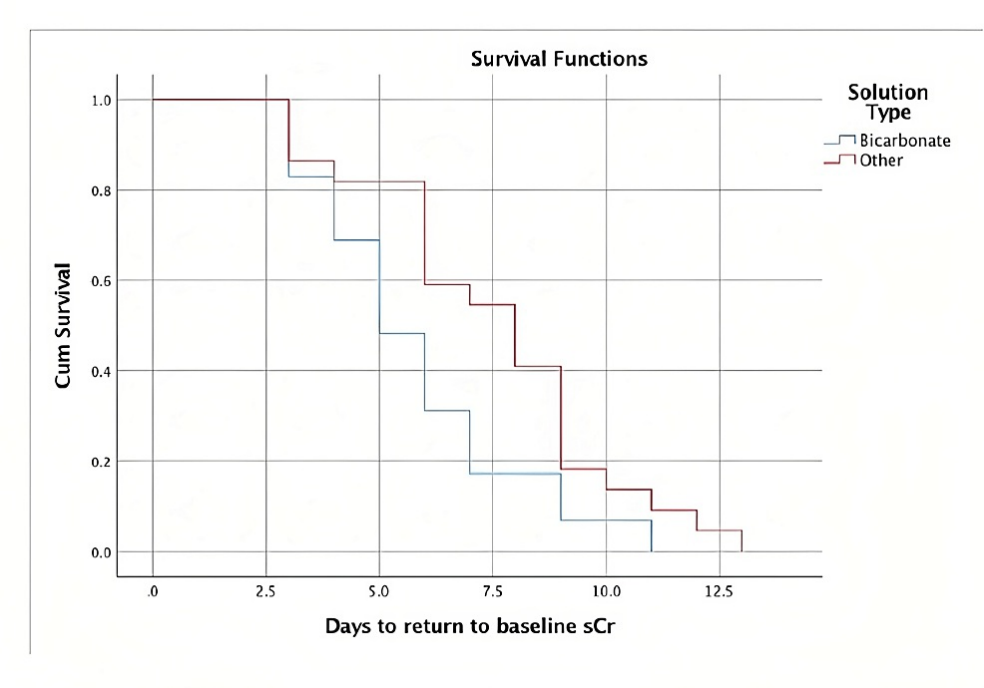
Time to return to a baseline sCr Time to return to a baseline sCr in patients who received bicarbonate-based solutions versus bicarbonate-free solutions.

Compared with the bicarbonate-free solution group, the time to return to the baseline sCr was also shorter in patients who received bicarbonate-based fluids (5.7 days (95% CI 4.9-6.6) vs. 7.5 days (95% CI 6.3-8.6); p < 0.022) (Figure 2).

The bicarbonate-based and bicarbonate-free groups received equal volumes of IV fluids (median (IQR), 1000 mL (500-2000 mL) vs. 1000 mL (1000-2000 mL); p = 0.90, respectively) with no differences found in daily urine output (Table 3). Hospital stay was shorter in patients who received the bicarbonate-based solution as compared to those who received bicarbonate-free solution (median (IQR), 7.65 (4-12) vs. 9.4 (4-14); p <0.001) and mortality at day 28 (3.4% vs. 13.3%; p <0.001).

## Discussion

In a heterogeneous population of hospitalized patients with varying stages of AKI, there was evidence for a faster resolution of AKI in patients who received the bicarbonate-based solution compared with the group of patients who received the bicarbonate-free IV solutions for resuscitation.

It is recognized that improving the hemodynamic status of patients and correcting intravascular volume deficit might have a positive effect on kidney function [12]. In hospitalized patients at risk of AKI or with established AKI, normal saline (0.9%) is one of the most commonly used crystalloid IV fluids for hydration or resuscitation. However, these types of solutions have supraphysiological concentration of chloride, which could induce metabolic acidosis. Recent studies have shown that the chloride content of solutions and the subsequent metabolic acidosis are linked to adverse effects on kidney function [4, 13, 14]. Possible mechanisms include the tubular chloride-induced renal vasoconstrictive effect [15] and activation of tubuloglomerular feedback, which in turn trigger arteriolar vasoconstriction, and mesangial contraction with an associated reduction in GFR [6, 16, 17]. The infusion of chloride may possibly also induce thromboxane release with associated vasoconstriction, interleukin production, increase renal endothelin-1 activity, and insulin resistance [18].

In a recent observational study among 30,994 patients undergoing major abdominal surgery, the use of normal saline increased mortality risk (5.6% vs. 2.9%; p < 0.001) and the risk of AKI requiring renal replacement therapy (4.8% vs. 1.0%; p < 0.001) as compared with the use of a balanced solution (Plasma-Lyte 148, Baxter Healthcare) [13]. Similarly, in a single-center, open-label, sequential study among 1,533 critically-ill patients, the change from solutions with high chloride content to solutions with low chloride content was associated with significant decrease in the risk of AKI (odds ratio (OR) 0.52, 95% CI 0.37-0.75, p < 0.01) and need of RRT (OR 0.52, 95% CI, 0.33-0.81, p = 0.004) [4]. In another study, among 12 healthy adult males, the investigators evaluated the effect of normal saline (154 mmol/L of chloride) and Plasma-Lyte 148 (98 mmol/L of chloride) on renal perfusion and blood flow velocity and found a significant decrease in mean renal artery flow velocity (p = 0.045) and renal cortical tissue perfusion (p = 0.008) from baseline in subjects who received normal saline. Normal saline infusion also decreased post-infusion urinary volume [19]. These studies suggest that solutions with high chloride content may negatively affect kidney function.

Our results are consistent with the above-mentioned studies in which there was a significant relationship between the use of high chloride content solutions and risk of AKI or its progression. In our study, we have observed a faster resolution of AKI with the use of a bicarbonate-based solution. The use of IV fluids with bicarbonate during AKI could provide several benefits, including 1) the rise of extracellular pH by sodium bicarbonate administration may directly improve cardiovascular function and tissue perfusion, and 2) since acute acidosis may impair cellular function, cause insulin resistance and promote interleukin production, the use of bicarbonate could potentially prevent kidney injury. Our study is unique as there are no randomized controlled trials that have explored the use of sodium bicarbonate in patients with established AKI.

It is important to note that there are few studies in the literature where no beneficial effects with the use of sodium bicarbonate or balance solutions [20, 21] were observed. For example, in one study, patients at risk of AKI (≥2 SRIS criteria, < 0.5 ml/kg/h for two hours of urine, and sNGAL ≥ 150 mg/L) were randomly assigned to receive sodium bicarbonate (0.5 mmol/kg diluted in 5% dextrose 250 mL over 1 hour, followed by an infusion of 0.2 mmol/kg/hour diluted in 5% dextrose 1000 mL over 47 hours) or sodium chloride (at same sodium bicarbonate dose). There were no differences in the incidence of AKI (*R*isk, *I*njury, *F*ailure,* L*oss, and *E*nd Stage Renal Disease (RIFLE) criteria) between patients who received bicarbonate as compared to those who received saline (11 vs. 10 patients; p = 0.93). Two patients in the sodium bicarbonate group required RRT versus none in the placebo group (p = 0.26) [20]. Of note, in this study, both solutions were hypertonic (equivalent to a sodium chloride 3%), and this could explain the presence of bicarbonate levels >30 mmol/L in four patients in the intervention group and chloride of >110 mmol/L in the control group [20]. More recently, Cho et al. [21] evaluated whether the use of sodium bicarbonate could prevent the development of AKI in patients with infective endocarditis following cardiac surgery. Using a similar protocol described above, 70 patients were randomly assigned to either 154 mEq/L sodium bicarbonate (0.5 mmol/kg loading dose over 1 hour, followed by 0.15 mmol/kg/h of a continuous infusion over 23 hours) or equivalent volumes of 0.9% sodium chloride for the same duration. The incidence of AKI was similar in both groups (29% vs. 23%; p = 0.6). The postoperative sCr increase in patients that received sodium bicarbonate was greater than in the saline group on day 2 (0.21 mg/dL vs. 0.06 mg/dL; p = 0.03) and on day 5 (0.23 vs. 0.06 mg/dL; p = 0.02). The opposite effects of sodium bicarbonate on renal function in both studies as compared with our study (no reduction on the incidence of AKI, and increased levels of sCr) could be explained by the amount of sodium bicarbonate used and the effect of alkalosis on renal vascular resistance [22]. The rapid influx of carbon dioxide_ _following sodium bicarbonate could also worsen intracellular acidosis [23], accelerating the influx of calcium and sodium, with subsequent cell swelling and renal tubular dysfunction [24].

Most previous studies have explored the relationship between the use of high chloride content fluids with the development of AKI or RRT requirement [4,5,25,26]. Our study is the first one to address the effect of a bicarbonate-based solution in patients with established AKI. A Cochrane intervention review could not find any randomized controlled trials that assessed the use of sodium bicarbonate supplements for the treatment of adults with AKI. Of note, the authors did not consider the inclusion of observational studies that investigated the use of sodium bicarbonate for AKI prevention [27]. The observation that the reduction in sCr is more pronounced and that the resolution of AKI was earlier in patients on bicarbonate solutions adds to the clinical implications of our observations.

The main limitation of our study is that it was not a blinded randomized trial. Thus, the unblinded nature of our study involving the use of several solutions that have compared balance solutions to chloride-rich crystalloids may introduce bias [4,28]. Secondly, we were not able to collect information on the acid-base status of individuals before and after the use of different types of solutions. The small sample size of our study is another limitation that did not allow us to adjust for different confounders. Finally, we were unable to examine the possible mechanisms that may have contributed to the faster recovery of kidney function.

## Conclusions

In patients with established AKI, the use of IV fluids with bicarbonate and low chloride content could improve renal function and accelerate renal recovery. This observational data supports the need for a randomized controlled trial to assess the use of bicarbonate-based solutions for the treatment of patients with established AKI.

## References

[1] Chertow GM, Burdick E, Honour M, Bonventre JV, Bates DW (2005). Acute kidney injury, mortality, length of stay, and costs in hospitalized patients. J Am Soc Nephrol.

[2] Coca SG, Yusuf B, Shlipak MG, Garg AX, Parikh CR (2009). Long-term risk of mortality and other adverse outcomes after acute kidney injury: a systematic review and meta-analysis. Am J Kidney Dis.

[3] Dellinger RP, Levy MM, Rhodes A (2013). Surviving sepsis campaign: international guidelines for management of severe sepsis and septic shock: 2012. Crit Care Med.

[4] Yunos NM, Bellomo R, Hegarty C, Story D, Ho L, Bailey M (2012). Association between a chloride-liberal vs chloride-restrictive intravenous fluid administration strategy and kidney injury in critically ill adults. JAMA.

[5] Yunos NM, Bellomo R, Glassford N, Sutcliffe H, Lam Q, Bailey M (2015). Chloride-liberal vs. chloride-restrictive intravenous fluid administration and acute kidney injury: an extended analysis. Intensive Care Med.

[6] Hashimoto S, Kawata T, Schnermann J, Koike T (2004). Chloride channel blockade attenuates the effect of angiotensin II on tubuloglomerular feedback in WKY but not spontaneously hypertensive rats. Kidney Blood Press Res.

[7] Bullivant EM, Wilcox CS, Welch WJ (1989). Intrarenal vasoconstriction during hyperchloremia: role of thromboxane. Am J Physiol.

[8] Kellum JA, Song M, Almasri E (2006). Hyperchloremic acidosis increases circulating inflammatory molecules in experimental sepsis. Chest.

[9] (2013). Chapter 1: Definition and classification of CKD. Kidney Int Suppl (2011).

[10] Chawla LS, Bellomo R, Bihorac A (2017). Acute kidney disease and renal recovery: consensus report of the Acute Disease Quality Initiative (ADQI) 16 Workgroup. Nat Rev Nephrol.

[11] Hoste EA, Maitland K, Brudney CS (2014). Four phases of intravenous fluid therapy: a conceptual model. Br J Anaesth.

[12] (2012). Section 3: Prevention and Treatment of AKI. Kidney Int Suppl (2011).

[13] Shaw AD, Bagshaw SM, Goldstein SL, Scherer LA, Duan M, Schermer CR, Kellum JA (2012). Major complications, mortality, and resource utilization after open abdominal surgery: 0.9% saline compared to Plasma-Lyte. Ann Surg.

[14] Reddi BA (2013). Why is saline so acidic (and does it really matter?). Int J Med Sci.

[15] Wilcox CS (1983). Regulation of renal blood flow by plasma chloride. J Clin Invest.

[16] Schnermann J, Ploth DW, Hermle M (1976). Activation of tubulo-glomerular feedback by chloride transport. Pflugers Arch.

[17] Salomonsson M, Gonzalez E, Kornfeld M, Persson AE (1993). The cytosolic chloride concentration in macula densa and cortical thick ascending limb cells. Acta Physiol Scand.

[18] Quilley CP, Lin YS, McGiff JC (1993). Chloride anion concentration as a determinant of renal vascular responsiveness to vasoconstrictor agents. Br J Pharmacol.

[19] Chowdhury AH, Cox EF, Francis ST, Lobo DN (2012). A randomized, controlled, double-blind crossover study on the effects of 2-L infusions of 0.9% saline and plasma-lyte® 148 on renal blood flow velocity and renal cortical tissue perfusion in healthy volunteers. Ann Surg.

[20] Schneider AG, Bellomo R, Reade M (2013). Safety evaluation of a trial of lipocalin-directed sodium bicarbonate infusion for renal protection in at-risk critically ill patients. Crit Care Resusc.

[21] Cho JS, Soh S, Shim JK, Kang S, Choi H, Kwak YL (2017). Effect of perioperative sodium bicarbonate administration on renal function following cardiac surgery for infective endocarditis: a randomized, placebo-controlled trial. Crit Care.

[22] Baretella O, Xu A, Vanhoutte PM (2014). Acidosis prevents and alkalosis augments endothelium-dependent contractions in mouse arteries. Pflugers Arch.

[23] Levraut J, Giunti C, Ciebiera JP, de Sousa G, Ramhani R, Payan P, Grimaud D (2001). Initial effect of sodium bicarbonate on intracellular pH depends on the extracellular nonbicarbonate buffering capacity. Crit Care Med.

[24] Velissaris D, Karamouzos V, Ktenopoulos N, Pierrakos C, Karanikolas M (2015). The use of sodium bicarbonate in the treatment of acidosis in sepsis: a literature update on a long term debate. Crit Care Res Pract.

[25] Yunos NM, Kim IB, Bellomo R (2011). The biochemical effects of restricting chloride-rich fluids in intensive care. Crit Care Med.

[26] Young P, Bailey M, Beasley R (2015). Effect of a buffered crystalloid solution vs saline on acute kidney injury among patients in the intensive care unit: the SPLIT randomized clinical trial. JAMA.

[27] Hewitt J, Uniacke M, Hansi NK, Venkat-Raman G, McCarthy K (2012). Sodium bicarbonate supplements for treating acute kidney injury. Cochrane Database Syst Rev.

[28] Annane D, Siami S, Jaber S (2013). Effects of fluid resuscitation with colloids vs crystalloids on mortality in critically ill patients presenting with hypovolemic shock: the CRISTAL randomized trial. JAMA.

